# The Complex Link and Disease Between the Gut Microbiome and the Immune System in Infants

**DOI:** 10.3389/fcimb.2022.924119

**Published:** 2022-06-15

**Authors:** Huan Zhang, Zhilin Zhang, Yiqun Liao, Wenjie Zhang, Dong Tang

**Affiliations:** ^1^ Clinical Medical College, Yangzhou University, Yangzhou, China; ^2^ Department of General Surgery, Institute of General Surgery, Clinical Medical College, Yangzhou University, Northern Jiangsu People’s Hospital, Yangzhou, China

**Keywords:** immune, infant, microbiome, maturation, diseases

## Abstract

The human gut microbiome is important for human health. The development of stable microbial communities in the gastrointestinal tract is closely related to the early growth and development of host immunity. After the birth of a baby, immune cells and the gut microbiome mature in parallel to adapt to the complex gut environment. The gut microbiome is closely linked to the immune system and influences each other. This interaction is associated with various diseases in infants and young children, such as asthma, food allergies, necrotizing colitis, obesity, and inflammatory bowel disease. Thus, the composition of the infant gut microbiome can predict the risk of disease development and progression. At the same time, the composition of the infant gut microbiome can be regulated in many ways and can be used to prevent and treat disease in infants by modulating the composition of the infant gut microbiome. The most important impacts on infant gut microbiota are maternal, including food delivery and feeding. The differences in the gut microbiota of infants reflect the maternal gut microbiota, which in turn reflects the gut microbiota of a given population, which is clinically significant.

## Background

Microorganisms have an extremely important impact on human health. In particular, the gut microbiome, consisting of trillions of microorganisms and millions of functional genes, have a mutually beneficial symbiosis with the host (Savage, 1977; Friedrich, 2013). The formation of intestinal micro-ecology in the early life plays a vital role in the healthy growth of life. The initial colonization of the infant gut by microorganisms lays the foundation for a lifelong, relatively stable adult microbiome (Houghteling and Walker, 2015). The host provides essential habitat and nutrients for the gut microbiome that provides the necessary nutrients to support the development of the metabolic system and the maturation of the immune system (Kau et al., 2011). Dietary changes during pregnancy and the early postnatal period can affect the colonization of the gut microbiome in infancy, affecting infant health and perhaps having long-term repercussions later in life (Cerdo et al., 2019). The gut microbiome directs the development and maturation of the immune system, which in turn regulates its composition, distribution and abundance (Garrett et al., 2010; Kayama et al., 2020). When the balance between the gut microbiome and the immune system is disturbed, it can lead to the development of a wide range of diseases (Elson and Alexander, 2015). Dysbiosis of the gut microbiome is closely linked to a number of diseases such as obesity (Turnbaugh et al., 2006), type 2 diabetes (Qin et al., 2012), hypertension (Yang et al., 2015), asthma (Arrieta et al., 2015), allergies (Metsala et al., 2013), necrotizing small bowel colitis (NEC) (Mai et al., 2011) and inflammatory bowel disease (IBD) (Frank et al., 2007; Kau et al., 2011). There is increasing evidence that the gut microbiome and the immune system can interact to have a significant impact on the growth and health of infants.

The intergenerational transmission of the gut microbiome can shape the biology of humans in kinship, leading to differences in their innate and adaptive immune systems and modulating their immunopathological status and risk of disease (Garrett et al., 2010). Systematic changes in the overall dietary consumption patterns of entire populations may lead to changes in the gut microbiota/microbiome, with consequent effects on the nutritional status and immune response of the host (Kau et al., 2011). It is important to understand the factors that influence and modify an individual’s microbiome at different life stages, with a focus on the early years of life (Kumbhare et al., 2019). In this review, we discuss the maturation and interaction of the infant gut microbiota and immune system. Finally, we also summarize infant diseases related to gut dysbiosis and immune system disorders and present our outlook.

## Maturation of Immune Cells and Gut Microbiome Parallel Each Other

Our gastrointestinal system is sterile or contains few microorganisms when we are born, and it is swiftly colonized by microbes from the outside world (Knoop et al., 2018). From birth to adulthood, gut microbial colonization in healthy individuals is characterized by a dynamic series of events that play critical roles in promoting gut homeostasis and stimulating normal development and responses of the immune system(**Table 1**) (Del Chierico et al., 2015; Dominguez-Bello et al., 2019). After birth, the fetus is suddenly exposed to an external environment rich in bacteria which immediately colonize the intestinal cavity with a large number of microorganisms, mainly Enterobacteria and Staphylococcus (Koenig et al., 2011; Del Chierico et al., 2015; Matsuki et al., 2016). CD4^+^ CD25^+^ Tregs are abundant and can perform their functions well due to microbial colonization. From 1-7 days after birth, the intestinal microbiome proliferates rapidly and in an orderly manner, forming a relatively mature microbial ecosystem dominated by Proteobacteria, Firmicutes, Bacteroidetes and Actinobacteria on the 7th day of birth (Bokulich et al., 2016). At the same time, neutrophils, the immune cells of the gut, increase exponentially, accompanied by the development of monocytes and macrophages, which reach maturity at the same time around the seventh day of birth (Ygberg and Nilsson, 2012). Gut microbial colonization and microbiota diversity lead to the induction of intestinal Tregs and IL-10, which are required to limit the induction of pro-inflammatory Th1 and Th17 cells (Geuking et al., 2011). There is evidence that conventionalization with a standard microbiota or monoclonalization with Bacteroides fragilis in the first 2 weeks of life can prevent abnormal accumulation of colonic iNKT cells and abolishes the increased susceptibility to colitis and asthma (Olszak et al., 2012; Gensollen et al., 2016). Cahenzli et al. demonstrated that microbial colonization early in life, rather than in adulthood, can keep IgE levels low and facilitate a reduction in susceptibility to orally induced allergic reactions (Cahenzli et al., 2013). After the age of 2 years, the abundance of Firmicutes and Bacteroidetes is greater than 90% of the total bacterial load, and the gut microbiome gradually develops to adult levels (Gosalbes et al., 2013; Martin et al., 2016). In summary, postnatal maturation of immune cells and gut microbiota occurs in parallel to adapt to the complex environment of the gut.

**Table 1:** Progression of the infant immune system and gut microbiome during the same period.

Period	Gut microbiome	Immune system
Before birth	Sterile, or contains few microbes	Immaturity and difficulty in coping with the external environment
Birth	Mainly Enterobacteria and Gram(+) cocci	The number of NK cells reach maximum number and CD4^+^ CD25^+^ Tregs are abundant and can function
1-7 days	Explosive proliferation of microorganisms	Neutrophils increase exponentially and monocytes and macrophages develop
7 days	Mainly Proteobacteria, Firmicutes, Bacteroidetes and Actinobacteria	Monocytes and macrophages mature
1 mouth	Mainly Firmicutes and Bacteroidetes	CD20^+^ cells maintain high levels
2 years	Significant total increase in bacteria and approaching adult levels	Immunity is more mature and gradually approaching adult levels

## Infant Gut Microbiome Promotes Immune System Maturation

The gut microbiome is critical for the immune system development, and its number and variety can influence the development of the intestinal immune system. Commensal microorganisms and their products can interact with immune cells to establish and maintain host tolerance while influencing innate and adaptive immune responses in early life (Li et al., 2014).

**Figure 1:**
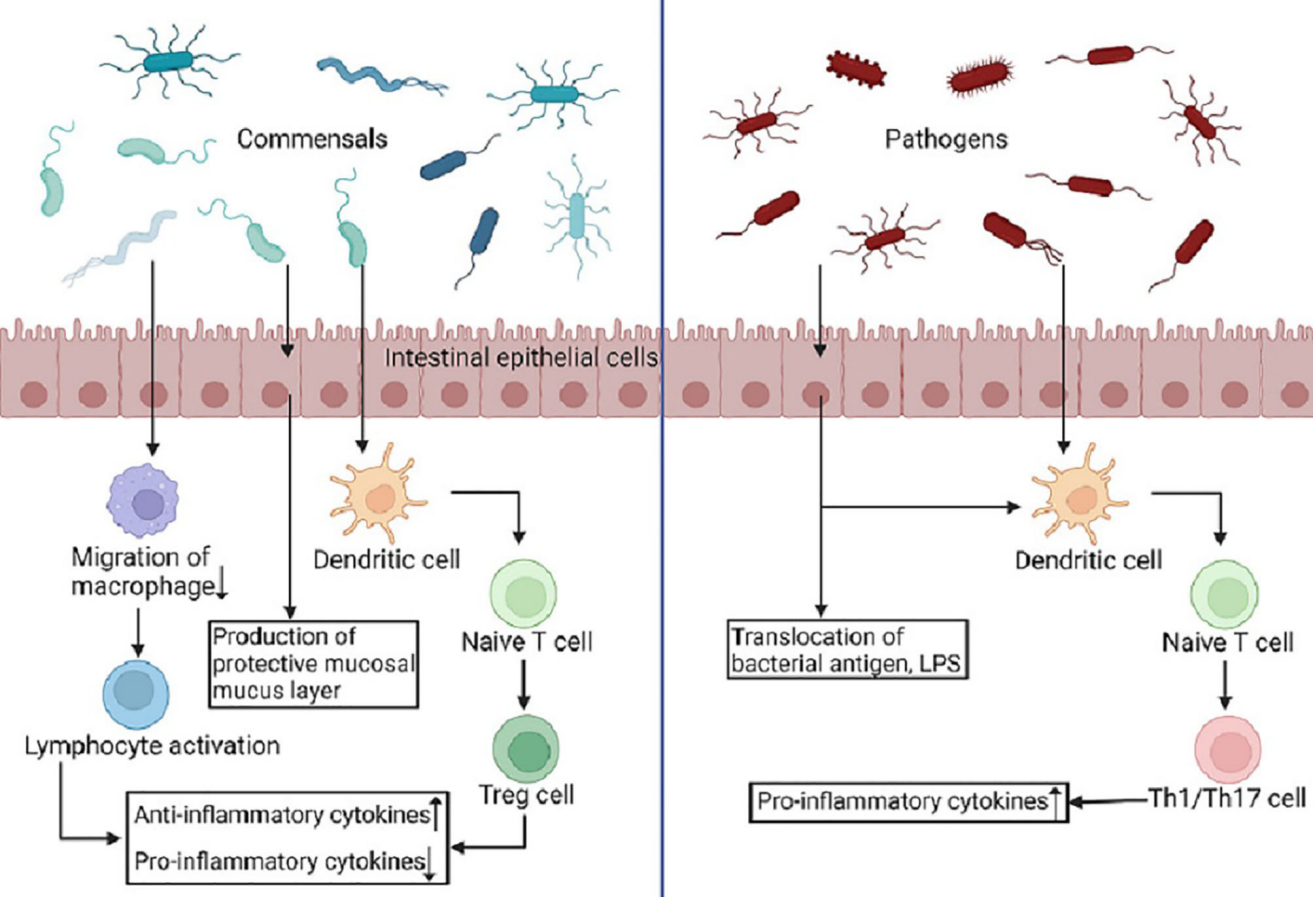
Commensals and pathogens produce different immune responses. PRRs identify MAMPs on pathogens and symbionts and are extensively expressed in intestinal epithelial cells, macrophages, and dendritic cells. Dendritic cells offer antigens to naive T cells as a result of commensals, and these cells differentiate into Treg cells, which emit anti-inflammatory cytokines and spread systemic and local tolerance. Simultaneously, phagocyte migration is inhibited, allowing microbial antigens to reach lymphoid tissue and increasing B- and T-cell activation. Conversely, pathogenic germs cause dendritic cells to secrete pro-inflammatory cytokines, which cause naive T cells to differentiate into Th1 and Th17 cells, resulting in a pro-inflammatory immune response. (Created in BioRender.com).

The development of the innate immune system, as well as the generation of immune cells, cytokines, and immunological factors, is influenced by the gut microbiome. The gut microbiome can exert pathogenic and protective functions through specific pattern recognition receptors (PRRs) and microbial pattern signaling pathways (MAMPs) and the subsequent immune responses (**Figure 1**) (Barko et al., 2018). Because different gram-negative bacteria have different lipopolysaccharide (LPS) alterations, their ability to trigger Toll-like receptors (TLRs) varies, not all bacteria will elicit the same immune response (Barko et al., 2018). Díazropero et al. applied *Lactobacillus salivarius CECT5713* and *Lactobacillus fermentum CECT5716*, both obtained from human milk, to bone marrow-derived macrophages, resulting in the induction of pro-inflammatory cytokine production by *Lactobacillus fermentum CECT5716* and, conversely, the promotion of interleukin-10 production by *Lactobacillus salivarius CECT5713* (Diaz-Ropero et al., 2007). Gram-negative bacterial peptidoglycan is recognized by nucleotide-binding oligomerization domain (NOD) 1 in intestinal epithelial cells, produces CCL20 and β-defensin 3, and directs B cell uptake into LTi in a cryptocurrency-expressing cluster -DC, thereby reducing sIgA (Maynard et al., 2012). Olzaked et al. demonstrated that natural killer T cells (iNKTs) accumulate in the colonic lamina propria and lungs of microbe-free mice, leading to increased incidence in models of inflammatory bowel disease (IBD) and allergic asthma (Olszak et al., 2012). The regulation of the expansion of iNKT cells was ascribed to reduced expression of the chemokine CXCL16 in the presence of microbiota. The diversity of the gut microbiome determines the degree of immunoglobulin E (IgE) in their adult bodies (Cahenzli et al., 2013). In atopic allergy illness and parasite immunity, IgE antibodies play a critical role (Gould and Sutton, 2008; Allen and Maizels, 2011). Early in infancy, a reduced IgA binding to gut bacteria was linked to an increased susceptibility to the development of asthma and allergies (Dzidic et al., 2017). The gut microbiome can facilitate the process of lymphocyte maturation and secretion of immunoglobulin A (IgA) (Bouskra et al., 2008). Bifidobacterium infantis supplementation inhibited T helper type 2 and T helper type 17 cell responses while stimulating interferon β production in breastfed babies, indicating the establishment of a positive regulatory immunological response (Henrick et al., 2021; Rachid et al., 2021). Beneficial bacteria induce apoptosis stimulation, reactive oxygen species synthesis and toll-like receptor signaling to control the inflammatory response and help establish innate immune defences and improve pathogen recognition (Kaplan et al., 2011; Jakaitis and Denning, 2014).

Therefore, the establishment of gut microbiome in early infancy has a crucial impact on the development of the immune system, its ability to fight off infections and also on its health in adulthood. Zeng et al. demonstrated the ability of gut microbes to induce immunoglobulin G (IgG) production in the organism, thereby inhibiting infection by pathogenic bacteria (Zeng et al., 2016). Schuijt et al. used *Streptococcus pneumoniae* to infect normal mice and mice with gut microbiome removed and found that the alveolar macrophages of the mice with gut microbiome removed had a reduced ability to phagocytose *Streptococcus pneumoniae* and a higher mortality rate, suggesting that gut microbes can enhance the function of major alveolar macrophages (Schuijt et al., 2016). Intestinal commensal bacteria can induce the secretion of inhibitory peptides, limit the inflammatory response and promote intestinal mucosal repair by blocking the recognition of TLRs to the intestine or by down regulating the NF-κβ pathway by blocking the degradation of NF-κβ inhibitors (Sampath et al., 2011). However, in immunocompromised hosts, the gut microbiome is not harmless, but may instead trigger an inflammatory response (Hooper et al., 2012). For example, the gut microbiome can contribute to the risk and pathogenesis of malnutrition by affecting nutrient metabolism and immune function (Kau et al., 2011). Cukrowska et al. showed that non-pathogenic *Escherichia coli* stimulates an immune response during intestinal colonization, but the intestinal mucosal immune response soon diminishes and tolerance forms due to the suppressive mechanisms (oral tolerance) (Cukrowska et al., 2001). Some symbiotic microbiomes are unable to express adhesion and invasion factors, or exhibit only low endotoxicity, and therefore cannot invade the intestinal mucus layer to elicit an immune response (Golenbock et al., 1991). Meanwhile, because the immune system of infants is relatively weakly regulated, exposure to antigens such as the gut microbiome is highly susceptible to lifelong or long-term tolerance, which means a specific non-responsive response to the gut microbiome.

In conclusion, commensal bacteria and the immune system can promote and favor each other and maintain the microecological balance of the gut. Instead, pathogens can disrupt the gut microbiome and stimulate the immune system to mount an inflammatory response. Imbalances in infant gut microbiome can lead to an incompletely developed the immune system in infants. Hence, abnormalities in the microbial composition of the gut of premature newborns may predispose them to postnatal failure in the evolution of key innate defences, leading to disease (Jakaitis and Denning, 2014).

## Infant Immune System Regulates the Composition of Gut Microbiome

The immune system influences the composition and abundance of the gut microbiome and can strengthen the epithelial barrier function in infants. The immune system of the intestinal mucosa consists of lymph nodes, lamina propria, and epithelial cells that form a physiological barrier that protects the integrity of the gut (Shi et al., 2017). Innate immune cells use a diversity of PRRs, including Toll-like receptors, NOD-like receptors and leptin-like receptors, to recognize conserved molecular patterns of microbes and signaling of these receptors may then act as developmental triggers for the gut microbiome (Fulde et al., 2018; Price et al., 2018; Dogra et al., 2021). IL-22 produced by innate lymphoid cells is in a position to induce hRegIIIα production in epithelial cells, which binds to bacterial peptidoglycan carbohydrates and kills Gram-positive target bacteria (Mukherjee et al., 2014; Shi et al., 2017). Kubinak et al. showed that the innate adaptor MyD88 in gut T cells is dependent on a signaling pathway and works with IgA to regulate the intestinal microbiome (Kubinak et al., 2015). IgA directly targets intestinal microbes and the absence of MyD88 signaling leads to an abnormal response of IgA antibodies and an imbalance in the intestinal microbiome, leading to inflammatory disease (Kubinak et al., 2015). However, interestingly, Wen et al. showed that non-obese diabetic mice lacking the specific pathogen-free MyD88 protein do not develop type 1 diabetes (Wen et al., 2008). sIgA prevents the adhesion of pathogenic microorganisms by forming complexes with their antigens and stimulating the secretion of mucus by cup cells in the mucosa of the respiratory and digestive tracts (Pulimood et al., 1998). Intestinal epithelial cells influence the composition of the gut microbiome by secreting antimicrobial proteins and alpha-defensins (Maynard et al., 2012). Mice with restricted NLRP6 expression in colonic epithelial cells showed an increased abundance of the bacterial phyla Bacteroidetes in the gut microbiome, as well as an increased susceptibility of intestinal inflammatory cells, thus inducing the development of colitis (Elinav et al., 2011).

During vaginal birth, the newborn is colonized with bacteria from the mother’s intestines and vagina, laying the foundation for a strong immune system (Milani et al., 2017). Infants born by cesarean section have a less complex gut microbiome and are less likely to be colonized than infants born by vaginal delivery (Milani et al., 2017). However, the lack of contact with the mother’s birth canal and inadequate exchange of intestinal microbiome may increase the risk of obesity, diabetes, and asthma in infants born by cesarean section (Algert et al., 2009; Li et al., 2013; Milani et al., 2017). Infants born by cesarean section require breastfeeding or formula feeding for normal and beneficial gut microbiome. In the early years of life, when the immune system is immature, breast milk provides infants with the necessary immune cells and immune related factors to fight infection (Goldman, 2007; Hanson, 2007; Riskin et al., 2012). Macrophages and leukocytes from breast milk can kill *enteropathogenic Escherichia coli, Giardia lamblia, Staphylococcus aureus, and Candida albicans*, thus contributing to the gradual recovery of infants who have been breastfed (Riskin et al., 2012). Breast milk contains compounds that may affect the immunity of newborns, including a group of oligosaccharides synthesised in the lactose gland, which is known as breast milk oligosaccharides (HMOs) (Bode, 2012; Plaza-Diaz et al., 2018; Singh et al., 2022). HMOs directly modulate the intestinal immune response, ensure enrichment of the gut microbiome and regulate microbial adhesion, thus protecting breastfed infants from microbial infections (Bode, 2012; Chichlowski et al., 2012; Singh et al., 2022). HMOs can stimulate the growth of beneficial microorganisms, mainly Bifidobacterium genus and some strains of Bacteroides and Lactobacillus (Sanchez et al., 2021).

Under normal conditions, the interaction between the immune system and the gut microbiome promotes the development of the infant gut and the immune system, enabling the infant gut to rapidly tolerate food antigens and preventing pathogenic bacteria from invading the mucosa and submucosa. Understanding the tight interplay between the gut microbiome, epithelial cells, and immune cells critical for maintaining gut homeostasis could facilitate advances in diagnostic and therapeutic approaches to various diseases.

## Gut Microbiome and Immune System Diseases of Infants

An abnormal microbiome can colonize an infant, impair immune system development after birth, and have long-term effects on health problems such as the development of allergies. Prenatal, inter-pregnant, or neonatal use of antibiotics can adversely affect the development of the neonatal gut microbiome and immune system, contributing to food allergies, asthma, necrotizing enterocolitis, obesity, and more in children (**Table 2**) (Milliken et al., 2019; Gao et al., 2021). All of these diseases have an overall delay in microbial maturation. Interventions for these disorders may include breastfeeding, changes to the infant’s diet and the use of probiotics (Bokulich et al., 2016; Mendez et al., 2019; Milliken et al., 2019).

**Table 2:** Diseases associated with the gut microbiome and their prevention in infants.

Disease	Gut microbiome	Prevention
Food allergy	Decreased production of butyrate by Clostridiae	Addition of Lactobacillus GG to formulae High fiber feeding
Asthma	Bacteroides fragilis subgroup and Clostridium coccoides subcluster XIVa species	Increase MACs and SCFA exposure during pregnancy
Necrotizing enterocolitis	Increase of Proteobacteria and decrease of Firmicutes and Bacteroidetes	Addition of HMOs to formula
Obesity	Increase of Clostridium cluster XIVa and Lactobacillus and decrease of Bifidobacterium, Aopobium and Escherichia coli	Addition of Lactobacillus and Bifidobacterium species to formula
Inflammatory bowel disease	Increase of Fusobacterium and adherent-invasive Escherichia coli and decrease of Bacteroides, Firmicutes, Clostridia, Bifidobacterium and Lactobacillus	Symbiotic formulations such as Lactobacillus GG and inulin, Bifidobacteria and fructoligosaccharides, and Bifidobacteria and lactobacilli with FOS or inulin.
Irritable bowel syndrome	Increase of Firmicutes: Bacteroidetes ratio and decrease of Bifidobacterium and Lactobacillus	Reduce intake of fermentable oligosaccharides, disaccharides, and monosaccharides and polyols Increased intake of short-chain inulin-type fructan

### Food Allergy

Changes in the microbial composition of the gut are one of the risk factors for food allergies. Hygiene hypothesis suggests that in the extended family, infection at an early age in children due to unhygienic contact with family members can prevent the development of atopic diseases (Strachan, 1989). Reduced family size and reduced opportunities for infection can lead to an increase in the development of atopic diseases (Strachan, 1989). Some bacterial strains as well as microbial diversity supports the maturation of regulatory T lymphocytes and promotes tolerance to food antigens, such as butyrate, a short-chain fatty acid (SCFA) produced from dietary fiber predominantly by Clostridia (Iweala and Nagler, 2019). Conversely, the misuse of antibiotics can disrupt microbiota diversity and thus promote allergy attacks (De Martinis et al., 2020). Tan et al. found that a high fiber diet changed the gut microbial ecology and enhanced SCFA release, especially acetate and butyrate, enhancing oral tolerance and avoiding food allergies in mice (Tan et al., 2016). Intestinal commensals can interact with DCs, macrophages and innate lymphocytes to promote IL-22 production, strengthen the barrier function of intestinal epithelial cells and prevent food allergy (Iweala and Burks, 2016). In a prospective clinical trial, Bernini Canini et al. showed that the addition of Lactobacillus GG to extensively hydrolyzed casein formula increased the tolerance rate to cow’s milk protein in infants with cow’s milk allergy (Berni Canani et al., 2012). Notably, prematurity and low birth weight were not linked with changes in the risk of developing food allergies during childhood (Liem et al., 2007).

### Asthma

Asthma is a chronic inflammatory airway disease characterized by reversible airway obstruction and hypersensitivity, generally thought to be caused by a combination of genetic and environmental factors (Nyce and Metzger, 1997; Abdel-Aziz et al., 2019). Penders et al. found that early colonization of *Clostridium difficile* was associated with all atopic outcomes, including asthma (Penders et al., 2007). The development of the gut microbiome in the first year of life may influence the development of childhood asthma, and adequate microbial maturation during this period may protect children at risk for asthma (Stokholm et al., 2018; Depner et al., 2020). Germ-free mice have been demonstrated to develop an immune defence system against allergic asthma if colonized as a newborn, but not if colonized as an adult (Olszak et al., 2012). The early microbial composition of preterm infants is disturbed compared to full-term infants, which increases their chances of having a disturbed immune system and makes them more susceptible to infections due to a lack of immune maturity (Bokulich et al., 2016; Kumbhare et al., 2019). Vael et al. showed that fecal colonization of *Bacteroides fragilis* subgroup or *Clostridium coccoides* subcluster XIVa subgroup within age 3 weeks is an early indicator of possible future asthma (Vael et al., 2011; Shi et al., 2017). Oral administration of *Bifidobacterium shortum* M-16V has been shown to upregulate transforming growth factor-β1 signaling and promotes intestinal Bifidobacterial colonization in preterm infants, reducing inflammatory and allergic reactions in infants (Fujii et al., 2006; Patole et al., 2014). The World Allergy Organization (WAO) guidelines for the prevention of allergic diseases recommend the use of probiotics to prevent eczema in non-exclusively breastfed infants at high risk of allergy (Fiocchi et al., 2015). Breastfeeding can facilitate the colonization of the infant’s gut with beneficial bacteria, such as *Bifidobacteria*, and prevents asthma in later childhood (Kull et al., 2010). However, in the case of mothers with asthma, breast milk does not have this effect, probably due to the low level of *Bifidobacteria* in the mother’s milk (Li et al., 2014). Furthermore, the mother’s diet and exposure to gut bacteria during pregnancy can also influence the development of the fetal immune system and subsequent asthma in the offspring (Daley, 2014; Gray et al., 2017; Ober et al., 2017). Induction mediated by fetal lung regulatory T cells, increased microbiota-accessible carbohydrates (MACs) and SCFA exposure during pregnancy can reduce the risk of asthma in offspring (Gray et al., 2017). Therefore, modulation of the infant gut microbiome through breastfeeding, prebiotics and others during pregnancy or early life may prevent the incidence of asthma in later life.

### Necrotizing Enterocolitis

Premature infants are susceptible to sepsis and necrotizing enterocolitis (NEC) due to a compromised intestinal barrier, immature immunity and a dysbiosis of the intestinal microbiota (Daley, 2014; Cuna et al., 2021). Increased relative abundance of Proteobacteria and decreased relative abundance of Firmicutes and Bacteroidetes characterize microbial dysbiosis in preterm infants prior to NEC (Pammi et al., 2017). Changes in the gut microbiome can lead to changes in host immunity and barrier function, which can contribute to NEC clinical signs and symptoms (Li et al., 2014). Human milk oligosaccharides (HMOs) are a specific type of non-digestible carbohydrates found in mother’s milk that aid in the formation of the infant gut microbiome, epithelial barrier, and immune system (Akkerman et al., 2019; Zhang et al., 2022). HMOs are of great importance in the prevention and treatment of NEC. In a single-center randomized control trial, the incidence of NEC was found to be significantly lower in preterm infants fed with formula containing HMOs compared to those fed with formula without HMOs, and the length of hospital stay was significantly shorter (Armanian et al., 2014). Austin et al. found that this may be explained by the disialyllacto-N-tetraose influence on TLR- and selectin-mediated inflammatory pathways in HMOs (Austin et al., 2019). Therefore, breastfeeding has a preventive and therapeutic effect on NEC to some extent. Because of the lack of microbial diversity in infant gut and its ease of control, the study of the infant gut microbiome has significant implications for the survival and prognosis of preterm infants at high risk of NEC.

### Antibiotics

Antibiotic abuse can lead to disruption of the infant gut microbiome, which can lead to an imbalanced immune response and eventually the development of many diseases in infants and young children. Epidemiological studies have established an association between the use of antibiotics in early infancy and the development of diseases such as obesity, diabetes and asthma in later life (Marra et al., 2006; Gasparrini et al., 2016). Nogacka et al. demonstrated that on day 7 after perinatal antibiotic administration, the proportion of Actinobacteria and Bacteroides in infant intestine decreased, while the proportion of Aspergillus and Firmicutes increased, resulting in a dysbiosis of the gut microbiome (Nogacka et al., 2017). Bokulich et al. found that antibiotic exposure disrupts microbial succession and can slow down the maturation of the microbiome (Bokulich et al., 2016). In addition, antibiotic exposure may cause an incomplete, yet stable and permanent, recovery of the microbiome, possibly leaving the recipient vulnerable to infectious diseases later in life (Dethlefsen et al., 2008; Gasparrini et al., 2016). Therefore, physicians must pay close attention to the serious consequences of antibiotic abuse, strictly control the indications for antibiotic use, and use antibiotics scientifically and rationally.

## Conclusions

There is growing evidence that the infant immune system and the gut microbiome are intimately connected, interact and mature together. Imbalances in the infant microbiome and disturbances in the immune system can also be causally linked to the development and progression of diseases. Theoretically, differences in the gut microbiome of infants at different stages and between individuals could reflect changes in the infant immune system, help define the human genetic evolution, reflect our changes in our eating habits, and enable disease prevention or new treatment strategies (Kau et al., 2011). However, techniques for the quantitative analysis of the innate and adaptive immune system are not yet fully developed, and biomarkers relevant to the infant gut microbiome, including those for mucosal-associated barrier function, are lacking. Furthermore, the current studies are still in the preclinical stage and the results are highly variable and uncontrollable, which still poses a challenge for clinical application. Therefore, there is a need to better understand the composition of the gut microbiome during early life stages, as well as the process of microbial community succession and evolution.

## Author Contributions

HZ and ZZ drafted the manuscript and counted and plotted the diagram and tables. DT critically revised the article for important intellectual content. All authors read and approved the final manuscript. All authors contributed to the article and approved the submitted version.

## Funding

This work was supported by grants from the Training Project of Key Talents of Youth Medicine in Jiangsu province, China [No. QNRC2016330], the Key disease standardization diagnosis and treatment project in Jiangsu province [NO. BE2015664], the Academic Science and Technology Innovation Fund for College Students [No. ×20180714], the Social Development-Health Care Project of Yangzhou, Jiangsu Province [No. YZ2018087], the Social Development Project of Yangzhou, Jiangsu Province [No. YZ2021075], the Graduate Research and Practice innovation Plan of Graduate Education Innovation Project in Jiangsu Province [NO. SJCX211644], and High-level talent”six one projects” top talent scientific research project of Jiangsu Province [No. LGY2019034]. The funding bodies had no role in writing the manuscript.

## Conflict of Interest

The authors declare that the research was conducted in the absence of any commercial or financial relationships that could be construed as a potential conflict of interest.

## Publisher’s Note

All claims expressed in this article are solely those of the authors and do not necessarily represent those of their affiliated organizations, or those of the publisher, the editors and the reviewers. Any product that may be evaluated in this article, or claim that may be made by its manufacturer, is not guaranteed or endorsed by the publisher.

## Abbreviations

IBD, Inflammatory bowel disease; iNKT, Invariant natural killer T; IgA, Immunoglobulin A; IgE, Immunoglobulin E; IgG, Immunoglobulin G; NEC, Necrotizing small bowel colitis; NOD, Nucleotide-binding oligomerization domain; PRRs, Pattern recognition receptors; MAMPs, Microbe‐associated molecular patterns; MACs, Microbiota-accessible carbohydrates; SCFA, Short-chain fatty acid; HMOs, Human milk oligosaccharides; TLRs, Toll‐like receptors; LPS, Lipopolysaccharide; WAO, World Allergy Organization.
